# Disparities in Care Outcomes in Atlanta Between Black and White Men Who Have Sex With Men Living With HIV: Protocol for a Prospective Cohort Study (Engage[men]t)

**DOI:** 10.2196/21985

**Published:** 2021-02-23

**Authors:** Patrick Sean Sullivan, Jennifer Taussig, Mariah Valentine-Graves, Nicole Luisi, Carlos Del Rio, Jodie L Guest, Jeb Jones, Greg Millett, Eli S Rosenberg, Rob Stephenson, Colleen Kelley

**Affiliations:** 1 Department of Epidemiology Rollins School of Public Health Emory University Atlanta, GA United States; 2 Division of Infectious Diseases Department of Medicine Emory University Atlanta, GA United States; 3 amFAR, the Foundation for AIDS Research Washington, DC United States; 4 Department of Epidemiology School of Public Health University at Albany, State University of New York Albany, NY United States; 5 Department of Systems, Population, and Leadership School of Nursing University of Michigan Ann Arbor, MI United States; 6 The Center for Sexuality and Health Disparities University of Michigan Ann Arbor, MI United States

**Keywords:** HIV, men who have sex with men, health disparities, viral suppression

## Abstract

**Background:**

The US HIV epidemic is driven by infections in men who have sex with men and characterized by profound disparities in HIV prevalence and outcomes for Black Americans. Black men who have sex with men living with HIV are reported to have worse care outcomes than other men who have sex with men, but the reasons for these health inequities are not clear. We planned a prospective observational cohort study to help understand the reasons for worse HIV care outcomes for Black versus White men who have sex with men in Atlanta.

**Objective:**

The aim of this study is to identify individual, dyadic, network, neighborhood, and structural factors that explain disparities in HIV viral suppression between Black and White men who have sex with men living with HIV in Atlanta.

**Methods:**

Black and White men who have sex with men living with HIV were enrolled in a prospective cohort study with in-person visits and viral suppression assessments at baseline, 12 months, and 24 months; additional surveys of care and risk behaviors at 3, 6, and 18 months; analysis of care received outside the study through public health reporting; and qualitative interviews for participants who experienced sentinel health events (eg, loss of viral suppression) during the study. The study is based on the Bronfenbrenner socioecological theoretical model.

**Results:**

Men who have sex with men (n=400) were enrolled between June 2016 and June 2017 in Atlanta. Follow-up was completed in June 2019; final study retention was 80% at 24 months.

**Conclusions:**

Health disparities for Black men who have sex with men are hypothesized to be driven by structural racism and barriers to care. Observational studies are important to document and quantify the specific factors within the socioecological framework that account for disparities in viral suppression. In the meantime, it is also critical to push for steps to improve access to care, including Medicaid expansion in Southern states, such as Georgia, which have not yet moved to expand Medicaid.

**International Registered Report Identifier (IRRID):**

DERR1-10.2196/21985

## Introduction

For men who have sex with men in the United States, each step of the HIV care continuum [1] is marked by racial disparities—those between non-Hispanic Black men who have sex with men and non-Hispanic White men who have sex with men [2]. Disparities for Black men who have sex with men manifest from systemic and structural racism, among many other health and social disparities [3]. Black men who have sex with men living with HIV have the least favorable care and treatment outcomes of all men who have sex with men [4]. Recent national HIV care continuum estimates reflect that among all US men who have sex with men, Black men who have sex with men as a group have the worst clinical outcomes, measured in terms of linkage to care (both within 1 and 3 months of diagnosis), retention in care, and viral suppression [4].

Despite increases in the number of Black men who have sex with men taking antiretroviral therapy and developing viral suppression, they are still less likely than White men who have sex with men to be prescribed and adhere to antiretroviral therapy and sustain viral suppression [5-7]. The US Centers for Disease Control and Prevention estimates that 41% of Black men who have sex with men compared to 59% of White men who have sex with men sustained viral suppression in 2014 [8]. A 2015 study [7] of selected infectious disease practices documented lower levels of viral suppression in Black non-Hispanic men who have sex with men (72%) than in White non-Hispanic men who have sex with men (91%). Surveillance estimates after 2014 have not been published for Black men who have sex with men specifically, although 2018 estimates of viral suppression among people in care for HIV in the US were 56% among Black people and 69% among White non-Hispanic people [9]. Black men who have sex with men were also estimated to be more likely to experience longer periods with viral loads >1500 HIV RNA copies/mL, an indicator of increased risk of transmission [8].

The southern US census region has the highest concentration of people living with HIV among men who have sex with men in the country [10], and Georgia is the historically most impacted state in both absolute number and prevalence rate of men who have sex with men living with diagnosed HIV [11]. Racial disparities in HIV between Black men who have sex with men and White men who have sex with men in the southern US are comparable to those seen at the national level [12]. Data collected from Black and White men who have sex with men in Atlanta, Georgia, showed an HIV prevalence of 43% among Black men who have sex with men compared to 13% among White men who have sex with men [12]. There were also racial disparities in CD4 count and STI prevalence as well as in rates of poverty, unemployment, and median income [12].

The National HIV/AIDS Strategy goals for 2016 to 2020 prioritize reducing racial disparities in HIV care and treatment [13], and the Ending the HIV Epidemic Plan for America recognizes the critical role of viral suppression in reducing HIV morbidity and in preventing HIV transmission [14]. Gaining a better understanding of the factors underlying racial disparities in HIV care and prevention, and addressing them through interventions, is critical for actualizing improvements both locally and nationally. The Ending the HIV Epidemic plan does not explicitly address health inequities by race but identifies a number of focus areas in the southern United States, including Atlanta, as priority areas, and sets ambitious national goals for reducing new HIV infections in the United States by 2025 [14]. Achieving these goals will not be possible without reducing transmissions among men who have sex with men, who comprise approximately two-thirds of new annual HIV diagnoses [4,15]. The strategies correctly identify treating all people with HIV as early as possible as a key component to achieve reductions in HIV infections [14].

Furthering our understanding of disparities in HIV treatment, care, and prevention between Black and White individuals requires new types of data collection. Although data on the HIV care continuum by race are available through surveillance data sources and in some clinical settings, the traditional means of depicting care continuum data are limited in that they use cross-sectional data to describe a longitudinal process [1]. People living with HIV have to enter clinical care to be included in the research, and once someone is lost to clinic follow-up, they are typically absent from the data set. Lapses in care are not well described. Clinic-based cohorts do not capture people who may change clinics or relocate [16]. Research using cross-sectional data has produced overestimates of care continuum trends [17]. Data on viral suppression, for example, primarily focus on a single viral load measure per patient in care within a 12-month period [17-19]. Research suggests that this tends to overestimate the percentage of patients with HIV and with stable suppressed viral load by as much as 16% [17]. Cross-sectional estimates have also been shown to underestimate the extent of racial disparities along the care continuum.

The limited longitudinal data we have suggest a widening of the racial disparity in retention in care and viral suppression over time. Colasanti et al [18] found that the racial disparity in rates of retention in care among Black and non-Black patients living with HIV did not exist when measured 12 months after initial observation, but the disparity became apparent when measured at a 24-month timepoint and continued to widen over time. They found a similar trend in viral suppression over time [18]. In short, existing data sources do not capture the myriad reasons for retention (or lack of retention) in care. There is a clear need to follow individuals longitudinally as they navigate the care continuum from diagnosis to viral suppression, taking into account both individual and structural factors that affect care and treatment engagement and outcomes.

**Figure 1:**
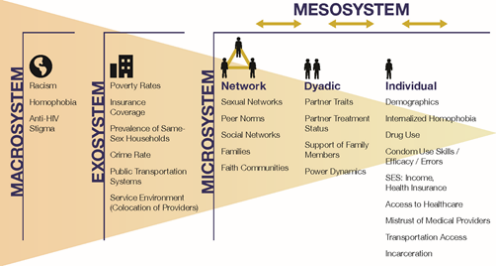
Socioecological model of factors potentially associated with worse HIV care outcomes for Black men who have sex with men living with HIV, adapted from Bronfenbrenner [20] and Baral [21].

This protocol describes Engage[men]t, a prospective cohort study of 400 Black and White men who have sex with men living with HIV in Atlanta, Georgia. The study aims to longitudinally examine factors associated with disparities in key HIV care and prevention indicators between Black and White populations. Key care indicators include lack of or delays in linkage to or retention in care, antiretroviral therapy nonadherence, and detectable viral load. Key prevention indicators include disclosure of HIV status to sex partners and condom use with susceptible sex partners. Advancing our understanding of the modifiable factors associated with these care and prevention indicators has the potential to inform the development of interventions that may improve care and prevention outcomes. We hypothesize a priori that there is no direct causal effect of race on care outcomes; rather, we postulate that the apparent associations between race and care outcomes are explained by indirect effects through intermediate individual- and community-level factors that may manifest because of systemic and structural racism. As such, our study is grounded in the Bronfenbrenner [20] ecological systems model, a framework situating the HIV care continuum disparities in individual, social, and cultural level influences and their relative impact on Black men who have sex with men and White men who have sex with men [21]. We chose to use the ecological systems model to frame this study because of data showing that disparities between HIV prevalence in Black and White populations are influenced by factors at multiple environmental levels, namely dyadic, network, and neighborhood levels (Figure 1) [2,12].

## Methods

### Design

The study design is a prospective, observational cohort of 400 men who have sex with men living with HIV in Atlanta, Georgia with 2 groups: Black men who have sex with men and White men who have sex with men. Recruitment of the cohort took place from June 2016 to July 2017. Participants were followed for 2 years with study assessments at baseline, 3, 6, 12, 18, and 24 months. In our mixed methods design, we sought to use multiple types of data and modes of inquiry to address concepts of triangulation and expansion [22]; other data sources included HIV surveillance data reported to the Georgia Department of Public Health, medical records from participants’ clinical encounters, and qualitative data collected through in-depth interviews with a subset of participants. Study inclusion and exclusion criteria are shown in Textbox 1. Black men who have sex with men with Hispanic ethnicity were excluded because we believed that the patterns of care outcomes and associated factors may be different for Black men who have sex with men who are Hispanic compared to Black men who have sex with men who are not Hispanic. The study was approved by the Emory Institutional Review Board (IRB00086663).

**Inclusion and exclusion criteria.**

**Inclusion criteria**

- Self-report positive HIV status, confirmed by HIV antibody screening at baseline
- Assigned male sex at birth
- Currently identifies as male
- Age ≥16 years
- Self-report single race to be Black or White
- Able to complete survey instruments in English
- Lives in the Atlanta metropolitan statistical area
- Had at least one male sex partner in the 12 months before the baseline interview

**Exclusion criteria**

- Participant is determined to not be living with HIV per study testing at baseline
- Participant is currently enrolled in another HIV prevention or treatment clinical trial
- Self-report Hispanic ethnicity

### Recruitment

Participant recruitment involved use of venue-based, web-based, or virtual and print recruitment methods. All 3 methods linked potential recruits to a web-based survey portal with a short description of the study and the opportunity to consent and complete a brief eligibility survey on a study tablet or personal electronic device. The eligibility surveys were hosted on a secure, Health Information Portability and Accountability Act–compliant server (administered by Survey Gizmo). Men were asked to leave their contact information at the end of the surveys so that study staff could follow up and schedule an enrollment (baseline) visit.

In-person recruitment occurred at gay bars and at community events with a large men who have sex with men or HIV focus such as gay pride. Events were selected for attendance based on venue-space time sampling [23], as previously implemented for other cohort studies of men who have sex with men in Atlanta [12]. At these events, potential recruits read a short description of the study and completed a brief eligibility survey on a tablet. Web-based recruitment used banner advertisements on social media sites, such as Facebook and Instagram, and on sex-seeking sites, such as Grindr and Bareback RT [24]. When possible, ad placement was targeted to male profiles with self-described Black or White race and an interest in other men, as these are among the study eligibility criteria. Potential recruits clicked on a banner advertisement that connected them to the study eligibility survey. Print recruitment included advertisements in gay magazines and on MARTA, Atlanta’s public transportation system. Advertisements included a URL for the study eligibility survey and also had an option for men to text a phone number and receive a reply text with a link to an eligibility survey. Some men were recruited from a broader panel screener for multiple studies, administered to men in similar settings. Small numbers of men were recruited from other studies, from peers, and via the research group’s website. All recruitment modalities had unique codes embedded in the eligibility survey URL to track recruitment sources.

Before completing the study’s eligibility survey, men provided informed consent by clicking on a box affirming their decision to continue and consent to be screened for the study. At the conclusion of the survey, men were informed if they were preliminarily eligible for the study. Preliminarily eligible men were given the opportunity to leave their contact information for follow-up. Men who were not eligible were also provided an opportunity to leave their contact information for possible enrollment in other current and future studies within the research group.

Potential recruits were rescreened by study staff and eligible men were scheduled for an initial baseline visit at one of 4 sites where study enrollment occurred. These sites included an HIV-focused community-based organization, a public HIV medical clinic, an office location in Decatur, Georgia and Emory University’s Rollins School of Public Health.

### Data Collection

#### In-Person Study Visits at Baseline, 12 Months, and 24 Months

Eligible men attended a baseline visit that lasted approximately 3 hours and included the following activities: (1) meeting with a study counselor to confirm study eligibility; (2) completion of intake forms and assessment of HIV care experience in the preceding 12 months; (3) completion of the study questionnaire using computer-assisted self-interview software; (4) collection of biological specimens; and (5) discussion with the study counselor about HIV care and treatment and prevention strategies with sexual partners, and referrals to HIV linkage and care services and other social services. The types of data collected and their relationship to the conceptual framework are shown in Table 1.

**Table 1:** Assessment methods and outcomes for study indicators of effective HIV care and prevention.

Indicators of		Assessment methods	Baseline and cohort outcomes	Sentinel event qualitative data
**Effective HIV care**				
	Linkage	Survey responses Medical record abstractions	First care visit within 3 months, if unlinked	Failure to have first visit in 3 months
	Engagement and retention	Survey responses Medical record abstractions Public health surveillance data	≥1 visit in 6-month window, if linked to care	Failure to have ≥1 visit in 6 months
	Adherence	Survey responses Laboratory assessment of antiretroviral medication concentrations in blood Medical record abstractions	No missed doses within previous week	Missed ≥1 doses within previous week
	Suppression	Survey responses Medical record abstractions Public health surveillance data	No detectable viral load in previous 6 months	Loss of suppression among previously suppressed
**Effective HIV prevention**				
	Disclosure of HIV status to sexual partners	Survey responses	Aim 1: Disclosure to all new partners in previous 6 months Aim 2: Disclosure to all current and new partners in previous 6 months	Previously diagnosed: Failing to disclose HIV status to a new partner before first anal intercourse Newly diagnosed: Not having disclosed HIV status to all ongoing anal intercourse partners within 3 months
	Condom use with susceptible sexual partners	Survey responses	Consistent and complete condom use with all HIV-negative or unknown anal intercourse partners in the previous 6 months, in light of preexposure prophylaxis use by partners and viral suppression status [25]	Unprotected anal intercourse with a susceptible (HIV-negative or HIV-unknown status) partner

The baseline questionnaire collected demographics as well as information about HIV care and treatment, hepatitis and other STIs, drug and alcohol use, HIV disclosure and condom use with anal sex partners, health care access and utilization (including use of Ryan White Care Act–supported services), mental health and other psychosocial influencers, housing, and transportation. Sexual health questionnaire elements built on a previously described questionnaire design to elicit partner-specific data [26]. Biological specimen collection involved testing for the following: HIV infection, viral load and CD4 count, antiretroviral medications, hepatitis C, urethral and rectal gonorrhea or chlamydia, syphilis, and heavy alcohol or nonprescription drug use.

The 12-month in-person visit was very similar to the baseline visit with the exception that participants had the option to complete the 12-month survey from home in advance of their visit, and there was a limited laboratory specimen collection. At the visit, if the participant had not completed the survey at home, they were asked to complete a short computer-assisted self-interview survey on their HIV care and treatment experiences since their baseline visit, they had a quick check in with the study counselor about any referrals they need for HIV and other supportive services, and they provided a urine specimen and had blood drawn to test for CD4 count, HIV viral load, heavy alcohol use (using a carbohydrate-deficient transferrin test) [27] and nonprescription drug use using iCup 10-Drug Panel Test Cup (BioScan Screening Systems Inc).

The 24-month in-person visit mirrored the 12-month in-person visit, with the exception that the laboratory specimen collection was expanded to include all tests from baseline except for testing for HIV infection status. Participants were compensated US $60 for their baseline and 12-month visits and US $75 for their 24-month visit.

#### Web-Based Surveys at Months 3, 6, and 18

At months 3, 6, and 18, we emailed participants with a URL to complete a web-based survey remotely. These surveys were similar to the ones completed at baseline, 12 months, and 24 months and collected updated demographics and longitudinal data on HIV care and treatment experiences, sexual behaviors, and drug or alcohol use. For each completed web-based survey, participants received a US $40 electronic gift card of their choice to either Amazon, Target, CVS, or Starbucks.

#### Individual In-depth Interviews

The prospective cohort study was supplemented by the collection of rich qualitative data on participants’ HIV care and treatment experiences, and the factors underlying racial disparities. We selected a subset of participants (n=21) to participate in a series of in-depth interviews at 6-month intervals over the course of the cohort study. Participant selection was based on a combination of survey data (from baseline and 3 months) and laboratory data. The survey and laboratory data were used to identify participants who had experienced sentinel events. Sentinel care events included incident lapse in care visits, antiretroviral therapy adherence problems, detectable viral load in a previously suppressed participant, lack of first care visit within 3 months of diagnosis, and lack of initial viral suppression within 6 months of diagnosis. Sentinel prevention events included a lack of discussion of seropositive status with a new or ongoing anal intercourse partner and lack of condom use with a susceptible partner. The goal was to sample participants for the individual in-depth interviews who had experienced a range of lapses in the continuum of care. Interviews utilized a timeline approach to capture clinical, social, and sexual life events over the course of a 6-month period before the interview [28,29]. The timeline created at the first in-depth interview covered the period 6 months prior to the date of the in-depth interview and was recreated at each successive interview for the past 6-month period. This participatory research method generated a visual tool that forms the foundation of questioning in the interview. For the first in-depth interview, the interviewer asked the participant to add the milestones of their engagement in HIV care to the timeline, serving to establish patterns of service use. Questions focused on barriers and facilitators to service use. At each interview, questions focused on recent engagement in HIV care, with participants marking these behaviors onto the timeline. Questioning sought to understand the context of engagement in HIV care, using the stem question “Tell me about what was happening in your life during (event in question)?” with specific probes for each of the distal and proximal factors. Respondents used stickers of various sizes and colors to represent domains of influence and perceived magnitude of influence. Respondents were also free to annotate the timeline with other issues.

Audio data from the in-depth interviews were recorded, transcribed, and deidentified. Timelines were scanned and turned into diagrams. Data were analyzed and coded in MAXQDA (version 18.2.5, Verbi GmbH). First, deductive codes were added to the transcripts to highlight key themes and guide comparisons, followed by inductive codes. Two staff members independently coded each transcript, with discrepancies in coding discussed and resolved at team meetings. Timeline diagrams were coded to develop phenotypes of patterns: analysis of phenotypes involved grouping timelines into similar sets or patterns, with a particular focus on identifying linkages across phenotypes. This allowed us to build concepts grounded in the data to explain phenomena observed and to identify phenotypes that may be specific to Black men who have sex with men or White men who have sex with men.

#### Medical Record Abstraction

We obtained permission for the release of medical records and abstracted the clinical records of a sample of our participants to augment the survey and laboratory data we collected and to gain a better understanding of the degree to which medical record data can be used to validate self-report survey data. Participants signed medical releases for all HIV clinicians they reported to us, and the releases were used by study staff to request copies of medical records. Clinical visits for the 1 year preceding and 2 years during study enrollment were abstracted.

#### HIV Surveillance Data

In an attempt to validate the quality of HIV care and treatment-related data from self-reported surveys and from medical record abstractions, and to obtain a fuller picture of HIV care outcomes, participants consented to the release of their reportable laboratory data to the study, and we requested individual-level HIV surveillance data from the Georgia Department of Public Health. By law, Georgia Department of Public Health receives all reports of HIV western blot tests, CD4 counts and viral load tests performed on patients in Georgia. On a biannual basis, we provided Georgia Department of Public Health with a list of names for all consenting participants, for whom we requested specific HIV surveillance data (eg, first HIV-positive test, CD4 count, or viral load in a date range). These data will allow us to assess the quality of our self-report, laboratory, and medical record data and provide a less biased data set for examining viral load outcomes.

### Planned Analyses

#### Cross-sectional (Baseline) Analyses

Using the baseline visit interview and laboratory data from participants, we will initially identify factors associated with key care and prevention outcomes. Factors in the theoretical model will be considered as exposures; these factors are summarized by level of the socioecological model (eg, individual, microsystem, exosystem) in Table 2. Our approach assumes that there is no true direct causal effect of participant race on each outcome, and that the apparent associations between the 2 are explained via indirect effects through intermediate individual- and community-level factors. We will assess these factors by adapting traditional mediation analysis [30] to a change-in-estimate epidemiologic modeling strategy [31,32].

For each HIV care and prevention outcome *Y*, we will consider logistic regression models of the form:


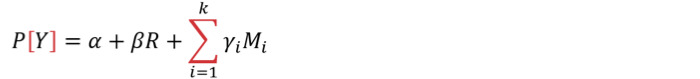


where *R* represents participant race and each *M*_i_ represents 1 of *k* factors in Table 2 as potential mediators of the association between *Y* and *R*. Because binomial models can be unstable, we will use predictive-margins adjusted *PR* to compare the adjusted *PR* for participant race in models with subsets of potential mediators, in a stepwise fashion [12,33,34]. A criterion of 10% change in the *PR* will be used to identify factors that meaningfully reduce the *Y*-*R* associations. The *PR* in the fully specified model, which controls for all potential mediators, indicates how many of the *Y*-*R* associations are explainable by the measured factors and the extent of any residual disparity. For identified mediators, we will examine the race-specific prevalence and the strength of association of each with the outcome *Y* to inform the highest-priority targets of intervention and research. Higher-order community and service environmental factors tend to be more distal to the *Y* of interest and operate through individual-, dyadic-, or network-level factors [35,36]. Therefore, these models will consider *M*_i_ at each explanatory level separately. This approach’s strength is that it allows for the identification of the factors that account for the racial disparities in HIV care among men who have sex with men, the degree to which they account for those disparities, and their relative roles within each racial group.

**Table 2:** Proposed measures in the theoretical model.

Type	Measures
Individual	Age, education, employment Health insurance coverage Incarceration history Stable housing Access to transportation Health literacy Health care perceptions and self-efficacy HIV treatment self-efficacy and optimism Depression, mental illness Experienced, perceived, internalized HIV stigma Experienced, perceived, internalized homophobia Experienced, perceived, internalized racism Drug use Condom efficacy, skills, errors Time since diagnosis HIV disease stage Social support
Microsystem	Dyadic Partner demographics, type, relationship strength Substance use Locations for meeting and sex Power and dynamics Network Peer norms for HIV care
Exosystem	Service environment Distribution of providers by service type Colocation of provider services Transportation options Community Poverty, insurance coverage, crime rates, Percentage same-sex households Community HIV stigma, gay stigma, racism

#### Statistical Power

Study power was estimated for the key outcome of HIV viral suppression, at the final analytic step of assessing the relationship of mediators with the outcome, among racial groups. We assumed n=160 per racial group, α=.05, power 80%, and race-specific suppression levels constrained to national estimates for HIV-diagnosed men who have sex with men by race [6,37,38]. Given 21% overall suppression among Black men who have sex with men, we anticipate 80% power to detect a binary mediating factor that is associated with a ≥60% reduction in viral suppression among Black men who have sex with men (ie, 30% vs 12% suppressed between the 2 levels of the mediator). Given 41% overall suppression among White men who have sex with men, we anticipate 80% power to detect mediators associated with a ≥40% reduction in viral suppression in this racial group.

Among those who had a suppressed viral load at the baseline visit, we will use the viral load data from each follow-up survey and study visit to identify time of loss of viral load suppression. For participants who experience a loss of viral load suppression, time of loss of viral load suppression will be defined as the midpoint between the first study visit at which their viral load was >40 copies/mL and the most recent study visit at which their viral load was suppressed. We will fit Cox proportional hazards models of the form:


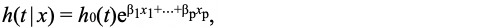


where *h*(*t*|*x*) represents the hazard at time *t* conditional on a set of covariates *x*, *h*_0_*t* represents the baseline hazard function, *p* signifies the number of parameters, and β values represent regression model parameters. We will estimate unadjusted hazard ratios for each of the variables considered. Time-varying measures will be used for variables that change over time (eg, substance use). Variables with statistically significant unadjusted hazard ratios will be included in a multivariable model to estimate the adjusted effect of each variable on time to viral load suppression.

#### Qualitative Data

Audio data from in-depth interviews will be recorded, transcribed and deidentified. Timelines will be scanned and turned into diagrams of sentinel events and their perceived influencers. Both the audio recordings and diagrams will be loaded into MAXQDA for coding and analysis. All interviewing and coding is team-based. We will implement a data handling and analysis plan based around principles of data reduction. We will use conduct grounded theory–based thematic analysis of the in-depth interview. Analysis of transcripts and diagrams began early in the data collection phase to identify emergent themes and we will continue iterative revision of the interview questions and probes. At weekly meetings, interviewers reviewed recent timelines and transcripts to identify codes and areas of questioning for future interviews. Through this process codebooks were developed that include a detailed description of each code, inclusion and exclusion criteria, and examples of the code in use. Deductive codes will be applied to the transcripts to highlight key themes and guide comparisons [39-41]. Principal deductive codes will be taken from the Bronfenbrenner model [20], and will include the codes *dyadic*, *network*, *community*, and *service environment*. Within each of these, subcodes will note the direction of influence as barrier or facilitator and the respondent’s perception of the magnitude of the influence. Next, inductive codes that represent newly emerging themes will be added to the transcripts.

Using this codebook, 2 researchers will independently code each transcript, and transcripts will be assessed for intercoder reliability using Cohen α [42]. If the κ statistic is found to be <0.80, discrepancies in coding will be discussed and resolved at team meetings. After all transcripts are coded, we will generate code frequency reports and look for patterns of code co-occurrence by key demographic characteristics (eg, age and race). Throughout this process, we will apply the rule of saturation; development of new codes will cease when no new themes are seen in the transcripts. In addition to the coding process, the timeline diagrams will also be coded to develop phenotypes of patterns observed. For example, one phenotype may be those with repeated similar sentinel events (eg, repeated refusal of antiretroviral medication), while another may be respondents who experience sentinel events at each stage of the treatment cascade. This involves grouping the timelines into similar sets or patterns.

Data analysis will involve generating frequencies of the codes and comparing the frequency of code occurrence across age and race, across phenotypes of sentinel event experiences, and across new or prevalent HIV-positive status. A particular focus of the analysis will be on identifying linkages across codes (eg, the extent to which those with major network influences also report service environment influences) and comparisons in patterns of these linkages by age and race and new versus prevalent positive HIV status within age and race. We will also compare code frequency between interviews with providers and cohort participants to examine whether there are differences in the perceptions of domains of influence on HIV care. This will allow us to build concepts grounded in the data to explain phenomena observed. The result will be the generation of a unique data set that illustrates racial variations in dynamic and multilevel influences on successful HIV treatment and care.

## Results

The study was approved in March 2016 and launched in June 2016. We enrolled 400 Black (n=206) and White (n=194) men in Atlanta, Georgia over the course of 1 year. Retention rates at 3, 6, 12, and 24 months were 95%, 95%, 87%, and 80%, respectively. A total of 53 qualitative interviews were conducted. The final study visit occurred in February 2019.

## Discussion

Disparities are a stubbornly persistent feature of the US HIV epidemic, and we have an increased understanding of the confluence of factors that drive these disparities. Race is a marker for a myriad of barriers to effective care, and observational cohort data offer the opportunity to understand which factors related to race mediate the relationship between Black race and worse HIV-related outcomes. That understanding can suggest priorities to reduce disparities. In other words, we know that associations with race and poor clinical outcomes are not causal, and our study sought to identify the patterns of associations that explain observed racial inequities. For example, our previous cohort of Black and White men who have sex with men in Atlanta [12] documented large disparities in the incidence of HIV between Black and White men who have sex with men and identified factors that mediated that relationship: lack of health insurance and higher prevalence of unsuppressed HIV infection in sex partners effectively explained all of the disparity in incidence [43,44]. These data supported calls (albeit unsuccessful ones) for expansion of Medicaid in Georgia. We have used the same approach to understand the factors that might explain the reasons for lower levels of HIV viral suppression for Black men who have sex with men.

We acknowledge that our study is subject to limitations. First, we are susceptible to selection bias if we recruit men whose patterns of exposures and viral suppression are not reflective of the underlying community of men who have sex with men in Atlanta. We attempted to mitigate this risk by using systematic methods to select venues, by not recruiting men who have sex with men living with HIV from care settings, and by screening high risk populations to identify newly diagnosed men who have sex with men to include a heterogeneous group of participants. We also anticipated the risk of selection bias if we had differential loss of men to follow up by important baseline characteristics. By conducting active follow-up, having shorter check in visits between annual visits, and maintaining multiple modes of contact, we were able to maintain high retention in the cohort. We attempted to minimize the risk of information bias in several ways. To reduce misclassification, we validated key outcomes (eg, HIV infection status, viral suppression, recreational drug use, problematic alcohol use) with biological measurements. We also supplemented our self-reported and annual viral load measurements by including all viral load measurements during the study period through public health surveillance data. We recognize that the social determinants of health we are considering as possible mediating factors in the association between Black race and lower levels of viral suppression are likely highly correlated with one another; in our analytic approach, we will assess mediation with a single factor at a time, and then use multivariable logistic regression to control for potential confounding.

Disparities in viral suppression for Black men who have sex with men are an inequitable health outcome that impacts the health of Black men who have sex with men living with HIV, and increases the risks of transmission for their partners. Identifying and addressing reasons for excess unsuppressed viral load in Black men who have sex with men is thus critical to improve the health and longevity of Black men who have sex with men living with HIV and to reduce levels of HIV incidence among men who have sex with men in the United States [10]. A further understanding of the factors associated with lack of viral suppression is critical to implementing programs to achieve national goals for effectively treating people living with HIV as part of the Ending the Epidemic: Plan for America [14]. We hope and anticipate that further elucidating the mechanisms for disparities in HIV care outcomes between Black and White individuals will help identify and advocate for targeted responses to improve viral suppression outcomes and reduce inequities. In the meantime, we already know from our work in Atlanta that Medicaid expansion is a critical step that can be taken now to benefit Atlanta men who have sex with men in terms of increasing preexposure prophylaxis uptake [45], reducing HIV incidence [43], and facilitating access to the HIV medications that can protect men living with HIV. We have sufficient data to call for an immediate expansion of access to health care for all Georgians, including Black men who have sex with men. According to our data, expanding access to health care would almost certainly reduce observed disparities in viral suppression.

## Appendix

Multimedia Appendix 1 National Institute of Health summary statement/peer reviews.

## Abbreviations

AIDS: acquired immunodeficiency syndrome
HIV: human immunodeficiency virus
RNA: ribonucleic acid
STI: sexually transmitted infection
