# Correction: Disparities in Care Outcomes in Atlanta Between Black and White Men Who Have Sex With Men Living With HIV: Protocol for a Prospective Cohort Study (Engage[men]t)

**DOI:** 10.2196/30020

**Published:** 2021-06-03

**Authors:** Patrick Sean Sullivan, Jennifer Taussig, Mariah Valentine-Graves, Nicole Luisi, Carlos Del Rio, Jodie L Guest, Jeb Jones, Greg Millett, Eli S Rosenberg, Rob Stephenson, Colleen Kelley

**Affiliations:** 1 Department of Epidemiology Rollins School of Public Health Emory University Atlanta, GA United States; 2 Division of Infectious Diseases Department of Medicine Emory University Atlanta, GA United States; 3 amFAR, the Foundation for AIDS Research Washington, DC United States; 4 Department of Epidemiology School of Public Health University at Albany, State University of New York Albany, NY United States; 5 Department of Systems, Population, and Leadership School of Nursing University of Michigan Ann Arbor, MI United States; 6 The Center for Sexuality and Health Disparities University of Michigan Ann Arbor, MI United States

In “Disparities in Care Outcomes in Atlanta Between Black and White Men Who Have Sex With Men Living With HIV: Protocol for a Prospective Cohort Study (Engage[men]t)” (JMIR Res Protoc 2021;10(2):e21985) the authors noted one error.

In the originally published manuscript, one author was inadvertently not displayed in the authorship list. Author Jodie L Guest was uploaded in the manuscript file, but inadvertently not entered in the online metadata. In the corrected manuscript, the order of authorship has been updated as follows:

Patrick Sean Sullivan; Jennifer Taussig; Mariah Valentine-Graves; Nicole Luisi; Carlos Del Rio; Jodie L Guest; Jeb Jones; Greg Millett; Eli S Rosenberg; Rob Stephenson; Colleen Kelley

The correction will appear in the online version of the paper on the JMIR Publications website on June 3, 2021, together with the publication of this correction notice. Because this was made after submission to PubMed, PubMed Central, and other full-text repositories, the corrected article has also been resubmitted to those repositories.

