# Supplementary material for: Disparities in Care Outcomes in Atlanta Between Black and White Men Who Have Sex With Men Living With HIV: Protocol for a Prospective Cohort Study (Engage[men]t)
Source: JMIR Res Protoc. 2021 Feb 23;10(2):e21985. doi: 10.2196/21985 (PMC7943338; doi:10.2196/21985)
Supplement: Multimedia Appendix 1 [file resprot_v10i2e21985_app1.pdf]

SUMMARY STATEMENT
(Privileged Communication)

PROGRAM CONTACT:
Rosemary McKaig
240-627-3214
rmckaig@niaid.nih.gov

Release Date: **12/29/2014**

Application Number: **1 R01 AI112723-01A1**

Principal Investigator

SULLIVAN, PATRICK SEAN DVM, PHD

Applicant Organization: EMORY UNIVERSITY

Review Group: **ZRG1 AARR-F (02)**
Center for Scientific Review Special Emphasis Panel
Member Conflict: AIDS and AIDS Related Research
AIDS - EXP. REV.

Meeting Date: **12/10/2014** ***RFA/PA:*** **PA13-302**
Council: **JAN 2015** ***PCC:*** **A27F**
Requested Start: **02/01/2015**
Dual IC(s): **MH**

Project Title: **Understanding disparities in effective HIV treatment and prevention: Involvement+**

SRG Action: **Impact Score: 27 Percentile: 13 #**
Next Steps: **Visit http://grants.nih.gov/grants/next_steps.htm**
Human Subjects: 30-Human subjects involved - Certified, no SRG concerns
Animal Subjects: 10-No live vertebrate animals involved for competing appl.
Gender: **3A-Only men, scientifically acceptable**
Minority: **1A-Minorities and non-minorities, scientifically acceptable**
Children: **1A-Both Children and Adults, scientifically acceptable**
Clinical Research - not NIH-defined Phase III Trial

Project Year	Direct Costs Requested	Estimated Total Cost
1	496,769	773,728
2	496,160	772,780
3	497,880	775,459
4	499,446	777,898
5	494,536	770,250
TOTAL	2,484,791	3,870,115

ADMINISTRATIVE BUDGET NOTE: The budget shown is the requested budget and has not been adjusted to reflect any recommendations made by reviewers. If an award is planned, the costs will be calculated by Institute grants management staff based on the recommendations outlined below in the COMMITTEE BUDGET RECOMMENDATIONS section.

1R01AI112723-01A1 Sullivan, Patrick

RESUME AND SUMMARY OF DISCUSSION: The applicant seeks to identify factors that explain the disparities in HIV infections among Black MSM compared to their White counterparts although the literature has not reported significant differences in the HIV risk behaviors of these populations. The applicant will leverage the infrastructure put in place for "InvolveMENt,“ this team's previous study of MSM, and will continue to involve community stakeholders. In this application, however, the investigators will focus on a prospective cohort of HIV-positive MSM to identify factors that can explain the racial disparities in HIV prevention and care. This very experienced and productive applicant has gathered a team of outstanding collaborators; together, there is no doubt they can carry out this project successfully. This resubmission was generally very responsive to previous critiques; its focus on MSM, a population well documented to be at the highest risk of HIV in this country is important. The plan to elucidate racial disparities between Black and White MSM is significant in that it will inform more effective, targeted interventions for Black MSM who continue to sustain the highest rate of HIV infections among MSM. The application has many strengths; it focuses on the care continuum; uses a mixed methods design, and will collect biomarkers, and the retention plan is very strong. These raise confidence this project will contribute important new information to the field. Enthusiasm for this strong application would have been much higher still, were it not for concerns with some aspects of the methodology. The most critical of these include a lack of justification for conducting STI tests and for interviewing providers; a concern that only including MSM not planning to move for the next two years will bias the sample; a lack of clarity as to whether information will be gathered about these MSM's partners, especially given data showing the critical role of partners in the disparities in HIV infection between Black and White MSM. Other, concerns included sample sizes that are potentially too small, the application's grounding in two theoretical frameworks that may be mutually incompatible, the absence of hypotheses to frame the results, and an insufficient focus on the multi-level components of the related factors, as purported. Despite these, however, there is no doubt this application will contribute substantially to generating new knowledge in understanding racial disparities in HIV/AIDS.

DESCRIPTION (provided by applicant): MSM are a key risk group in the United States and are disproportionately impacted in terms of HIV prevalence and incidence. For decades, black/white disparities in HIV prevalence and incidence have been recognized. Much of the dialogue about disparities has focused on what might cause disparities in new HIV infections, and we recently completed an NIMH-supported R01 (InvolveMENt) to examine reasons for black/white HIV disparities in an HIV/STI incidence cohort of black and white MSM in Atlanta. An early, important finding from that study is that HIV-negative black MSM, while having comparable levels of individual risk behaviors, have a much greater chance of having a sex partner with a detectable HIV viral load – thus black MSM are at higher risk for acquiring HIV, despite comparable individual risk behaviors. But disparities extend beyond risks for infection: black MSM may fare less well in the treatment cascade because of later access to care, lower adherence to antiretroviral therapy (ARV), mistrust of providers, or, perhaps, stigma and discrimination. Recent national estimates have documented consistent racial disparities at each step of the HIV treatment cascade, culminating in a 30% lower likelihood of viral suppression among black non-Hispanic, compared to white non- Hispanic HIV-positive individuals. This pattern has been observed in the state of Georgia. In a Lancet systematic review in 2012, Millet et al. illustrated that the racial disparities after HIV infection are profound for MSM: black MSM were found to be less likely to initiate ARVT, less likely to adhere to therapy, and less likely to be virally suppressed. Further, this analysis suggested that structural factors, such as education, poverty, and lack of employment, played key roles in care disparities. Thus, we propose to build on our previous MSM cohort in Atlanta, to identify the factors that are prospectively associated with racial disparities in effective HIV care and prevention. The proposed work will build on the infrastructure, community relationships, multilevel conceptual framework and multidisciplinary team assembled for our previous grant, but will shift focus from HIV-negative MSM to those living with HIV. The design will be a prospective cohort of HIV-positive black and white MSM in Atlanta, with rich laboratory, survey, and qualitative data collections. Results

will help provide deeper understanding of why disparities in care outcomes occur, and will provide actionable data for the development of interventions to reduce disparities in effective HIV care.

PUBLIC HEALTH RELEVANCE: Profound disparities in effective HIV care exist for black MSM compared to white MSM; the reasons are most likely related to structural and community factors. A cohort study of HIV-positive black and white MSM is proposed to further understanding of these disparities, and suggest possible interventions.

CRITIQUE 1:

Significance: 2
Investigator(s): 1
Innovation: 1
Approach: 2
Environment: 1

Overall Impact: This revised application proposes: Aim 1, to prospectively examine factors temporally associated with incident ineffective (and incident effective for Aim 2) medical care and prevention among MSM previously diagnosed (and newly diagnosed for aim 2) with HIV in Atlanta; Aim 3, to understand modifiable reasons for key missed opportunities for HIV care and prevention, by using nested, prospective qualitative data collections regarding “sentinel care events” or “sentinel prevention events. This is a highly meritorious, methodologically complex and ambitious study, with multiple sources of data that will generate a great deal of information. The study is highly descriptive, and although there is a theoretical explanatory model, there are no specific hypotheses. Thus, it is not completely clear what is the potential for explaining the ways in which health disparities relate to negative outcomes. Enthusiasm for impact is diminished by attempting to explain health disparities through comparisons between Black and White MSM rather than by specific mechanisms that exacerbate and perpetuate those disparities.

1. Significance:

Strengths

- Proposed aims are of significance. Findings will provide information to pursue causal models in future studies.

Weaknesses

- Black-White comparison is not of significance; it is well documented that disparities between these two groups exist.
- The applicants state that racial comparisons will help develop culturally appropriate interventions. Many culturally appropriate interventions have been developed without racial comparisons, but rather, by examining and understanding variability within racial groups.

2. Investigator(s):

Strengths

- Very accomplished PI with great research experience. Capable of recruiting the samples and of carrying out ambitious data collection. He has an excellent record of delivering results.
- Strong research team, mainly with biomedical expertise. Addition of Mr. Millet strengthens cultural competence of team.

Weaknesses

- None observed

3. Innovation:

Strengths

- The use of nested, prospective, mixed methods qualitative data collections regarding sentinel care events and sentinel prevention events is highly innovative.
- A prior reviewer questioned the innovation of examining cascade of care. The applicant has clarified that most studies have used cross-sectional data, whereas this study will use temporal, prospective data to examine the dynamics of the cascade of treatment from an ecological perspective.
- Qualitative component, as described in this application, is innovative.

Weaknesses

- None observed.

4. Approach:

Strengths

- Numerous strengths of this application; hard to list them all to do justice to application.
- Excellent use of preliminary data to develop recruitment plan; it seems feasible to identify 80 undiagnosed infections, which is the most challenging group to identify. However, it may be more challenging to recruit an equal number of Black and White participants given lower incidence in the latter group.
- Two-year follow-up with assessments will yield valuable information. Strong retention plan.
- In-depth interviews to follow up sentinel cases are very well described and promise to be a model for other researchers.
- Use of CASI, triangulation of in-depth interviews, biological data, medical chart abstraction.

Weaknesses

- There are no specific hypotheses guiding quantitative analyses. It seems that the same conceptual model will be used for Black and White MSM. Given that the end point is viral suppression, as written, it seems findings will reiterate once again that there are racial disparities. The opportunity to understand how different factors intervene and interact to create disparate outcomes within Black MSM could be missed.
- Conducting the analyses and interpreting results from so many sources of data can be unwieldy. Stepwise regression is not the most elegant way for analyzing the data. If there were more precise hypotheses, better analytic methods could be used.
- Unclear why HIV screening will be done at 24 months; they are all living with HIV (Table 2). Similarly, no justification for STI testing is provided.
- Minor: No questions about sexual behavior or adherence in measures section (Table 3), but presumably they are in survey as per text in application.
- No justification for medical providers' interviews. As proposed, it might simply reflect the extent to which medical providers agree with Bonfenbrenner's model.

- Unclear if there is sufficient power in the sample of 80, half white, half black, to detect the race by time interaction. There is a risk that there will not be enough variability in the sample of 80.

5. Environment:

Strengths

- Emory University is an excellent environment to carry out the aims of the study.

Weaknesses

- None observed.

Protections for Human Subjects:

Acceptable Risks and/or Adequate Protections

- No concerns; adequate protections.

Data and Safety Monitoring Plan (Applicable for Clinical Trials Only):

Not Applicable (No Clinical Trials)

Inclusion of Women, Minorities and Children and not IRB Exemption #4.

- Sex/Gender: Distribution justified scientifically
- Race/Ethnicity: Distribution justified scientifically
- Inclusion/Exclusion of Children under 21: Including ages < 21 justified scientifically
  - Only men, all ages, justified scientifically.

Vertebrate Animals:

Not Applicable (No Vertebrate Animals)

Biohazards:

Not Applicable (No Biohazards)

Resubmission:

- A concern brought up by previous reviewer may have not been completely addressed; nevertheless, the knowledge that this study will yield is invaluable. "Not clear why a need exists to verify previously documented prevalent racial disparities in previously diagnosed White and Black MSM".

Budget and Period of Support:

Recommend as Requested:

- The resources used to recruit a White sample could have been used to recruit a much larger Black sample, which would allow more robust statistical analyses, including SEM. This is

particularly important for previously undiagnosed infections, because if all 80 were Black other more important questions could be addressed.

- No budget for Mr. Millet, who says his contribution is inkind. How much effort will he be able to devote?

Additional Comments to Applicant (Optional):

  - I recommend deleting interviews to health care providers as they are and use those resources to increase Black sample size.

CRITIQUE 2:

Significance: 2
Investigator(s): 1
Innovation: 4
Approach: 3
Environment: 1

Overall Impact: Previous studies that have been carried out to understand Black/White disparities in care, treatment, and prevention among Black MSM have largely been cross sectional. The current study, in Atlanta with HIV+ Black and White MSM, attempts to use a prospective analysis to examine factors that explain incident lack of success in the care continuum. The authors seek to use survey methods, chart extraction, biomarkers and qualitative data to identify multi-level factors that explain the lack of initiation and retention in medical care. They also seek to identify modifiable targets along the care continuum using an analysis of sentinel care events. The team has substantial success in this area of work previously, the environment is outstanding, and the applicants have adequately responded to most of the previous reviewer comments, and have made clear attempts to bolster the impact of the current application. While they have improved the application, there are a few missed opportunities: inclusion of partners, restrictive inclusion criteria concerning stating that one will not move in the next two years, and lack of analysis of masculinity in the model or the measures limits the impact somewhat. In addition, the application still is not clear on whether it is truly multi-level—many indicators appear to be on the individual level or are about perceptions of the neighborhood level despite previous findings in the researchers' own team that indicate the importance of partner and neighborhood effects. Finally, previous reviewers did note the importance of intervention development, and the team did not take this into account all that much aside from the identification of some perceptions of modifiable impact—that wasn't an entirely strong response, although it is responsive.

1. Significance:

Strengths

- There are not that many papers nor funded projects carried out on HIV+ Black MSM that seek to understand Black/White disparities in care and that identify the factors that explain lack of success along the care continuum
- All too many cross-sectional studies are carried out when focused on the care continuum—very few longitudinal
- Nuanced use of qualitative data to examine sentinel care and prevention events is likely to uncover modifiable domains

- Solid changes have been made from the last revision indicating that the authors have taken reviews seriously and have more fully delineated what is and what is not known about HIV care disparities among Black and White MSM

Weaknesses

- Unclear work on providers is included in the qualitative analysis-HOW would providers be included in this study? How should they be? This was not a strong component of the proposal.
- Lack of inclusion of partners and information about partners limits the usefulness given the importance of partners noted in the background information
- Level of analysis appears to be focused at multiple levels but measures are often at the individual level—the need for multi-level analysis is made very clear in this application but it is not fully brought to fruition

2. Investigator(s):

Strengths

- This team has an outstanding background in this area of study, in this region, with excellent and long term success in recruiting and retaining study samples

Weaknesses

- None noted

3. Innovation:

Strengths

- While we do know that Black MSM fare poorly in the care continuum, we do not have enough information to intervene due to a lack of longitudinal data and a lack of nuanced understanding of the mechanisms through which health care disengagement occurs. This fills an important gap
- Temporal ecological lens is critical—far too many studies are focused at the individual level

Weaknesses

- While the chart is very helpful on page 77 to show the lack of previous studies and funded research in this area of study, the authors did not make it entirely clear what we do know about the other studies focused on HIV+ MSM who are Black (aside from Millet's findings of structural factors being important and Sullivan & Rosenberg finding that couple level factors mattered). Thus, reviewers are not entirely able to evaluate the innovation. Nine studies are listed in the chart and 3 are discussed.

4. Approach:

Strengths

- Use of biomarkers, survey, medical record abstraction and qualitative work strengthen the application.
- Reasonable changes have been made in the application that Indicate a real responsiveness to previous reviews, including removing restrictive inclusion criteria from the last round

Weaknesses

- The lack of inclusion or information gathered on partners seems like an important omission given the importance of partners that was highlighted in the background information (e.g. less discussion of HIV status and partner less likely to be virally suppressed)
- There was no discussion of masculinity in the theoretical model, the literature review, the measures, or the analysis despite the fact that men frequently do not seek care due to ideals of strength, invulnerability, and more that are particularly relevant to Black MSM
- The requirement that MSM must not be planning on moving within the next 2 years limits the sample to those who are more economically well off and stable. This biases the sample in a direction that would yield far less helpful information focused on resolving problems found along the care continuum. After all, Black MSM are far more transient and economically disenfranchised
- Unclear if the authors have maximized the analytical utility of a multi-level model—not only whether they are examining whether 1 level affects another but also the model didn't seem to be truly capitalizing on/adequately measuring levels beyond individual?

5. Environment:

Strengths

- This is outstanding.

Weaknesses

- None Noted

Protections for Human Subjects:

Acceptable Risks and/or Adequate Protections

Data and Safety Monitoring Plan (Applicable for Clinical Trials Only):

Not Applicable (No Clinical Trials)

Inclusion of Women, Minorities and Children and not IRB Exemption #4.

- Sex/Gender: Distribution justified scientifically
- Race/Ethnicity: Distribution justified scientifically
- Inclusion/Exclusion of Children under 21: Excluding ages < 21 justified scientifically

Vertebrate Animals:

Not Applicable (No Vertebrate Animals)

Biohazards:

Not Applicable (No Biohazards)

Budget and Period of Support:

Recommend as Requested:

CRITIQUE 3:

Significance: 2
Investigator(s): 2
Innovation: 3
Approach: 4
Environment: 1

Overall Impact: This is a prospective study designed to better understand why Black MSM experience reduced outcomes in terms of HIV care compared to White MSM. The study moves beyond individual-level factors to include structural and community factors. The study team is strong, well experienced, and productive. They are building on previous work in Atlanta.

1. Significance:

Strengths

- MSM are disproportionately affected by HIV.
- Black MSM carry further disproportionate burden.

Weaknesses

- None Noted

2. Investigator(s):

Strengths

- This is a very strong team that is well equipped to conduct this study.

Weaknesses

- Mr. Millett does not have dedicated effort; thus, it is difficult to judge his true involvement with this research.

3. Innovation:

Strengths

- Longitudinal study
- Mixed methods

Weaknesses

- Prior research documents the many challenges of Black and White MSM, as well as Black and White MSM, in regard to HIV care engagement.
- What are particularly needed are effective interventions to increase Black MSM engagement in HIV care.
- Use of theory is not an innovation.

4. Approach:

Strengths

- Well written application.
- Methodology is strong with the exception of a qualitative component.

Weaknesses

- The investigators suggest that they will use grounded theory but also say they are using a "deductive approach" based on Bronfenbrenner. These 2 approaches are not compatible. Grounded theory is designed for inductive theory development.
- How findings can provide insight to interventions is asserted but not described. How do the investigators suppose (as examples) the findings might be useful? This point seems particularly important when we think about what we already know about disparities versus what we need to do to reduce and eliminate them.

5. Environment:

Strengths

- Fine

Weaknesses

- None noted.

Protections for Human Subjects:

Acceptable Risks and/or Adequate Protections

Data and Safety Monitoring Plan (Applicable for Clinical Trials Only):

Not Applicable (No Clinical Trials)

Inclusion of Women, Minorities and Children and not IRB Exemption #4.

- Sex/Gender: Distribution justified scientifically
- Race/Ethnicity: Distribution not justified scientifically
- Inclusion/Exclusion of Children under 21: Including ages < 21 justified scientifically
- It is not clear why this does not include other minority populations such as Hispanic MSM. This is not addressed.

Vertebrate Animals:

Not Applicable (No Vertebrate Animals)

Biohazards:

Not Applicable (No Biohazards)

Resubmission:

- This submission is does not adequately address concerns identified in the previous review, most notably implications for intervention and the qualitative approach that remains less rigorous.

Budget and Period of Support:

Recommend as Requested:

THE FOLLOWING SECTIONS WERE PREPARED BY THE SCIENTIFIC REVIEW OFFICER TO SUMMARIZE THE OUTCOME OF DISCUSSIONS OF THE REVIEW COMMITTEE, OR REVIEWER'S WRITTEN CRITIQUES, ON THE FOLLOWING ISSUES:

PROTECTION OF HUMAN SUBJECTS: ACCEPTABLE

INCLUSION OF WOMEN PLAN (G3A): ACCEPTABLE

INCLUSION OF MINORITIES PLAN (M1A): ACCEPTABLE

INCLUSION OF CHILDREN PLAN (C1A): ACCEPTABLE

COMMITTEE BUDGET RECOMMENDATIONS: The budget was recommended as requested.

Ad hoc or special section application percentiled against "Total CSR" base.

NIH has modified its policy regarding the receipt of resubmissions (amended applications). See Guide Notice NOT-OD-14-074 at http://grants.nih.gov/grants/guide/notice-files/NOT-OD-14-074.html. The impact/priority score is calculated after discussion of an application by averaging the overall scores (1-9) given by all voting reviewers on the committee and multiplying by 10. The criterion scores are submitted prior to the meeting by the individual reviewers assigned to an application, and are not discussed specifically at the review meeting or calculated into the overall impact score. Some applications also receive a percentile ranking. For details on the review process, see http://grants.nih.gov/grants/peer_review_process.htm#scoring.

MEETING ROSTER

The roster for this review meeting is displayed as an aggregated roster that includes reviewers from multiple Center for Scientific Review Special Emphasis Panels of the AARR IRG for the 2015/01 council round.
This roster is available at:
http://www.csr.nih.gov/SummaryStatementRoster/AARR201501.pdf
